# Potential changes in bacterial metabolism associated with increased water temperature and nutrient inputs in tropical humic lagoons

**DOI:** 10.3389/fmicb.2015.00310

**Published:** 2015-04-15

**Authors:** Vinicius Scofield, Saulo M. S. Jacques, Jean R. D. Guimarães, Vinicius F. Farjalla

**Affiliations:** ^1^Department of Ecology, Biology Institute, Universidade Federal do Rio de Janeiro, Rio de JaneiroBrazil; ^2^Post-Graduate Program in Ecology, Universidade Federal do Rio de Janeiro, Rio de JaneiroBrazil; ^3^Post-Graduate Program in Ecology and Evolution, Federal University of Juiz de Fora, Rio de JaneiroBrazil; ^4^Institute of Biophysics Carlos Chagas Filho, Universidade Federal do Rio de Janeiro, Rio de JaneiroBrazil; ^5^Laboratorio Internacional en Cambio Global, Rio de JaneiroBrazil

**Keywords:** environmental changes, bacterial metabolism, bacterial growth efficiency, tropical aquatic ecosystems, coastal lagoons, water temperature

## Abstract

Temperature and nutrient concentrations regulate aquatic bacterial metabolism. However, few studies have focused on the effect of the interaction between these factors on bacterial processes, and none have been performed in tropical aquatic ecosystems. We analyzed the main and interactive effects of changes in water temperature and N and P concentrations on bacterioplankton production (BP), bacterioplankton respiration (BR) and bacterial growth efficiency (BGE) in tropical coastal lagoons. We used a factorial design with three levels of water temperature (25, 30, and 35°C) and four levels of N and/or P additions (Control, N, P, and NP additions) in five tropical humic lagoons. When data for all lagoons were pooled together, a weak interaction was observed between the increase in water temperature and the addition of nutrients. Water temperature alone had the greatest impact on bacterial metabolism by increasing BR, decreasing BP, and decreasing BGE. An increase of 1°C lead to an increase of ~4% in BR, a decrease of ~0.9% in BP, and a decrease of ~4% in BGE. When data were analyzed separately, lagoons responded differently to nutrient additions depending on Dissolved Organic Carbon (DOC) concentration. Lagoons with lowest DOC concentrations showed the strongest responses to nutrient additions: BP increased in response to N, P, and their interaction, BR increased in response to N and the interaction between N and P, and BGE was negatively affected, mainly by the interaction between N and P additions. Lagoons with the highest DOC concentrations showed almost no significant relationship with nutrient additions. Taken together, these results show that different environmental drivers impact bacterial processes at different scales. Changes of bacterial metabolism related to the increase of water temperature are consistent between lagoons, therefore their consequences can be predicted at a regional scale, while the effect of nutrient inputs is specific to different lagoons but seems to be related to the DOC concentration.

## Introduction

Changes in climate and biogeochemical cycles are among the most important effects of human alterations to ecosystems (Rockström et al., 2009). For instance, nitrogen deposition, and phosphorus loads related to sewage disposal are affecting natural aquatic ecosystems worldwide (Schindler and Vallentyne, 2008). Increases in air temperature and changes in rain patterns are also expected in most regions (IPCC, 2013). For the highly populated Brazilian Southeast in particular, an increase in the average daily temperature and dramatic changes in the intensity and periodicity of rainfall events are expected for the next century (Marengo et al., 2010; PBMC, 2013). These changes will impact the amount of energy and matter flowing into aquatic ecosystems by increasing water temperature and augmenting the input of nutrients from drainage areas, with consequences for the structure and functioning of aquatic ecosystems (Roland et al., 2012). Furthermore, in tropical aquatic ecosystems, synergy between climate-driven impacts and changes in the input of inorganic nutrients is expected, mainly in highly populated areas (Roland et al., 2012). Thus, studies integrating different environmental changes into the same experimental design are necessary to better understand the effects of synergistic interactions on the functioning of aquatic ecosystems.

Bacterioplankton play an essential function in the transfer of energy and materials in aquatic ecosystems. Bacterioplankton respiration (BR) mineralizes large amounts of organic carbon substrates, thereby contributing to CO_2_ saturation in aquatic ecosystems, whereas bacterioplankton production (BP, i.e., secondary production) is an important source of energy for higher trophic levels through the microbial loop food chain (Fenchel, 2008). The ratio of carbon incorporated into bacterial biomass to total carbon assimilated by bacteria (bacterial growth efficiency, BGE) has been used to evaluate whether and where bacteria act as a carbon source or sink in aquatic ecosystems (for a review, see del Giorgio and Cole, 1998). Several environmental factors regulate BGE in aquatic ecosystems, and any changes in these environmental factors would alter bacterioplankton metabolism, which, in turn, would alter bacterioplankton function in aquatic ecosystems. For instance, low concentrations of N and P limit BP in aquatic ecosystems (e.g., Granéli et al., 2004; Smith and Prairie, 2004; Haubrich et al., 2009) but BR appears to be less affected in oligotrophic aquatic ecosystems. Low BGE is expected in aquatic ecosystems with high C:N and C:P ratios, and the input of inorganic nutrients in oligotrophic waters should increase BGE. Rising water temperature increases the permeability of the cell membrane to carbon substrates, stimulating both BP and BR (Van de Vossenberg et al., 1999; Mansilla et al., 2004). However, higher temperatures could also decrease some enzymatic reactions, increasing the metabolic cost of maintenance and cellular repair and consequently decreasing BGE (Hall and Cotner, 2007). The effect of temperature on BGE can also be mediated by nutrient concentrations, but this is not a general response pattern. For instance, some studies reported a negative relationship between BGE and temperature in nutrient limiting conditions (e.g., Kritzberg et al., 2010), others reported a negative relationship between BGE and temperature when nutrients were abundant (e.g., Hall and Cotner, 2007; Berggren et al., 2010), while others found little or no effect of temperature on BGE, and suggested that BGE was primarily regulated by the availability of nutrients (e.g., López-Urrutia and Morán, 2007; Lee et al., 2009). Most studies on the effects of temperature on bacterial metabolism were performed in temperate ecosystems, but those effects could be more dramatic in tropical systems, where organisms are already in their optimum temperature conditions (Deutsch et al., 2008). In addition, most of these studies failed to disentangle the effects of temperature and nutrients on bacterial metabolism; this article aims to fill this gap in the literature evaluating the main and interactive effects of water temperature and nutrient additions on bacterial metabolism in tropical aquatic ecosystems.

Tropical coastal lagoons of the Brazilian Southeast have several characteristics that make them suitable ecosystems for the evaluation of how changes in environmental condition affect bacterial metabolism. First, these lagoons provide a natural gradient in environmental conditions (for a review, see Esteves et al., 2008). Inorganic N and P concentrations are low, and oligotrophic and dystrophic conditions prevail, though some effects of cultural eutrophication on nutrient availability have also been observed in lagoons located near major cities (Esteves et al., 2008). Water temperature varies synchronously in these lagoons (Caliman et al., 2010), and daily changes of more than 10°C have been observed in the shallowest lagoons (depth <1 m; Farjalla et al., 2005). Second, climate- and human-driven impacts on these lagoons are predicted to increase during this century (Esteves et al., 2008; Roland et al., 2012; PBMC, 2013). For instance, local population triplicate in the last few years and should double in the next decade driven by the development of the offshore oil industry, and there is good a 1:1 relationship between population growth and cultural eutrophication of coastal aquatic ecosystems in the region (Esteves et al., 2008; Borges et al., 2009). Climate prediction indicates an increase of 0.5–1.0°C through 2040 and of 2–3°C between 2041 and 2070 (PBMC, 2013). Third, bacterioplankton metabolism has been extensively studied in these lagoons (e.g., Farjalla et al., 2002, 2005, 2009a). Previous studies have found that BP is usually limited by the availability of inorganic P, though N limitation and N and P co-limitation were also observed (Farjalla et al., 2002). The quality of bulk Dissolved Organic Carbon (DOC) favors BR over BP, and BGE is typically low in these lagoons (Farjalla et al., 2002, 2009a). Under high temperature conditions (above 40°C), the water temperature regulates bacterial metabolism (Farjalla et al., 2005).

In this study, we evaluated both the main and interactive effects of water temperature increases and inorganic nutrient additions on the bacterial metabolism of tropical coastal lagoons in the Brazilian Southeast. Based on previous results, we expected (i) an increase in BP and BGE after inorganic nutrient additions, (ii) a decrease in both BP and BR with higher temperatures, (iii) synergistic effects between temperature increase and inorganic nutrient additions on bacterial metabolism. We established a full-factorial design in which water temperature and inorganic nutrients were manipulated and BP, BR, and BGE were evaluated following microcosm incubation. The increase in water temperature had a consistent effect on bacterial metabolism across all lagoons, increasing BR and decreasing both BP and BGE. The effects of nutrient additions varied among lagoons and seemed to be related to the concentration of DOC in the systems. We concluded that (i) different environmental stressors impact bacterial processes at different scales, and (ii) the temperature increase predicted for the next century would alter bacterioplankton function in these lagoons, diverting more carbon to bacterial catabolism and CO_2_ production and/or less carbon to bacterial biomass and higher trophic level through the microbial loop.

## Materials and Methods

### Study Area

The study was conducted in the Cabiúnas, Carapebus, Comprida, Amarra-Boi, and Atoleiro shallow coastal lagoons, all located in Restinga de Jurubatiba National Park in the coastal region of the Brazilian Southeast (-22° 17′ 30′′, -41° 41′ 30′′). These lagoons constitute a natural gradient in DOC concentration varying from less than 12 to more than 110 mg L^-1^ (**Table 1**). More than 90% of the total DOC is composed of allochthonous humic substances mostly derived from an impermeable soil layer rich in organic matter (Farjalla et al., 2009a). Chlorophyll-a concentrations are low, as is the availability of nitrogen and phosphorus (**Table 1**). Despite being located on the coast, salinity is low as is the pH (**Table 1**), which reflects the major contribution of humic-rich freshwater to these systems. Other limnological features can be found in Caliman et al. (2010), and a detailed map of the region and the study lagoons can be found in Laque et al. (2010).

**Table 1:** Abiotic conditions in the studied coastal lagoons during bacterial samplings in January/2009.

	pH	Salinity	DOC (mM)	DIN (μM)	DIP (μM)	Chlorophyll-a (μg L^-1^)
Cabiúnas	6.68	0.6	0.95	12.86	1.64	2.35
Carapebus	7.43	3.6	1.37	20.71	1.00	3.22
Comprida	4.07	0.1	4.18	37.86	1.54	2.16
Amarra-Boi	3.71	0.2	7.28	60.71	1.58	0.48
Atoleiro	3.43	0.2	9.18	43.57	1.85	0.92

*DOC, Dissolved Organic Carbon; DIN, Dissolved Inorganic Nitrogen; DIP, Dissolved Inorganic Phosphorus. See below information about the analytical methods.*

*pH was obtained using a pH meter (Analion PM 608) and salinity was measured using a portable Thermosalinometer multi-functional probe (YSI-30). For DOC, DIN, and DIP analyses, water samples were filtered through 0.7-μm glass fiber filters (Φ = 25 mm, GF/F, Whatman). DOC concentration was determined using a Pt-catalyzed high-temperature combustion method with a TOC-5000 Shimadzu Carbon Analyzer. DIN concentrations were calculated as the sum of *N*-nitrate and *N*-ammonium concentrations, which were, in turn, analyzed using a Flow Injection Analysis System (FIA – Asia Ismatec; Zagatto et al., 1980) and spectrophotometry after applying blue indophenol (Koroleff, 1978), respectively. DIP concentrations were determined using the molybdenum blue reaction (Golterman et al., 1978). Chlorophyll-a concentrations were measured through ethanol extraction and spectrophotometry (Nusch and Palme, 1975).*

### Sampling and BP, BR, and BGE Evaluations

Water samples were collected on three alternate days from the central point of each lagoon in January/2009. Each sampling day was treated as a block in the ensuing analyses (see Statistical Analysis below). For sampling, we used 5-l polyethylene bottles previously washed with 10% HCl and rinsed with deionized water. The samples were taken to the laboratory and filtered through 0.7-μm glass fiber filters (Φ = 47 mm, Macherey-Nagel GF-3) to remove particles and larger organisms. All sampling and filtering procedures were performed in approximately 6 h. Filtered water samples were divided among 20-mL glass vials previously washed with 10% HCl and rinsed with Milli-Q water.

A full factorial design was established for each lagoon, with four levels of addition of two different nutrients (nutrient treatment) and three temperature manipulations (temperature treatment). The nutrient treatment consisted of N addition (addition of KNO_3_, 50 μM final concentration), P addition (KH_2_PO_4_, 5 μM final concentration), N and P addition (additions of KNO_3_, 50 μM final concentration, and KH_2_PO_4_, 5 μM final concentration) and a control with no nutrients added. Added nutrients represented an increase from ambient conditions (control treatments) of 82 to more than 300% for nitrogen and 270–500% for phosphorus. The temperature treatment included incubations at 25, 30, or 35°C in BOD incubation chambers. The average water temperature is 25°C in the studied systems (see Caliman et al., 2010 for review) but values up to 40°C were observed in sporadic drought events in summer months (Farjalla et al., 2005), which should occur more frequently according to projections for the region (Roland et al., 2012). Six replicates were performed for each treatment in each lagoon, yielding a total of 360 bacterial cultures. All incubations were performed in the dark for 48 h. Previous tests and other studies showed bacterial cultures established from water samples from these lagoons responded rapidly to nutrient additions or temperature changes. For instance, daily changes in bacterial production related to changes in the water temperature were observed in another lagoon located in the same area (Farjalla et al., 2005), while bacterial metabolism responded in 24–48 h after nutrient additions in Cabiúnas, Carapebus, and Comprida lagoons (Farjalla et al., 2002).

Bacterioplankton production was evaluated in each bacterial culture at the beginning and end of the 48-h incubation using the ^3^H-leucine incorporation method (Kirchman et al., 1985) and trichloroacetic acid (TCA) protein extraction (Smith and Azam, 1992), as modified by Miranda et al. (2007). An intracellular isotopic dilution factor of two was used in the calculations (Simon and Azam, 1989). Incubations were performed in the dark for 45 min with 20 nM of ^3^H-leucine (specific activity 150 Ci mmol^-1^). The concentration of 20 nM of ^3^H-leucine was previously established from saturation curves (unpublished data). We set up negative controls by adding 90 μL of TCA before starting the incubations. After the incubation, ^3^H-leucine incorporation was stopped by the addition of 90 μL TCA. Bacterial protein was extracted by washing with 5% TCA and 80% ethanol. After protein extraction, we added a liquid scintillation cocktail [EcoLite(+)^TM^] to each sample, and the samples were radio-assayed by liquid scintillation counting (Beckman LS – 6500) after 2 days in the dark to reduce the chemiluminescence. Bacterial protein production was converted to BP (g C 1^-1^ h^-1^) using a C:protein ratio of 0.86, according to Wetzel and Likens (1991). BP ratio unit was further converted to μM C h^-1^ to comparisons with other nutrient concentrations.

Bacterioplankton respiration was assessed by measuring oxygen consumption in each bacterial culture at the beginning and end of the 48-h incubation using an oxygen micro-sensor (Clark-type sensors, OX-N, Unisense) connected to a picoamperimeter (PA 2000, Unisense), following Briand et al. (2004). This approach is highly accurate, stable and has a low response time, and has been widely used to study bacterial metabolism (steering sensitivity <2%, response time <10 s; Briand et al., 2004). A respiratory quotient of 1.2 was used to convert oxygen measurements to carbon values (Berggren et al., 2012). Based on the results of BP and BR, we calculated BGE according to the formula BGE = BP/(BP+BR) (del Giorgio and Cole, 1998).

### Statistical Analyses

The main and interactive effects of each factor on BP, BR, and BGE were assessed using linear models (LMs), linear mixed effect models (LMMs), or non-linear mixed effect models (NLMMs). First, to assess the global effects of treatments and their interactions we pooled together the data of all lagoons. We used LMM and NLMM with additions of N and P set as categorical explanatory factors, temperature as the continuous explanatory variable, sampling blocks nested within lagoons as the random categorical factor, and BP, BR, and BGE as the dependent variables. Because using NLMM had not improved the results (verified by using the Akaike’s information criterion), we kept and showed only the LMM results. The Gaussian family (link = “identity”) was used to fit the model. To meet test assumptions, BR and BGE were log-transformed. Because only the temperature treatment showed consistent effects on bacterial metabolism in this first analysis (see results below), we (i) ran LMs relating BP, BR, and BGE with the water temperature, and (ii) ran different LMMs for each lagoon to analyze the effects of N and P addition treatments and their interaction on each system separately, searching for common responses to nutrient additions among lagoons. In this case, N and P were set as categorical explanatory factors, sampling blocks nested within temperature treatments as the random categorical factor, and BP, BR, and BGE as dependent variables. Again BR and BGE data were log-transformed to meet test assumptions and the Gaussian family (link = “identity”) was used to fit the model. Finally, as we observed two different groups of lagoons with similar types of bacterial responses to N and P additions (low and high DOC concentration ecosystems; low <1.5 μM C, high >4.0 μM C), we re-ran the latter analysis, pooling together the bacterial data for each group. We used contrast analysis to distinguish significant differences between the levels of N and P additions. Tests were performed using the “nlme” (Pinheiro et al., 2014) and “multcomp” (Hothorn et al., 2008) libraries in the R statistical software (R Development Core Team, 2014), and an α criterion level of 0.05 was used.

## Results

Bacterioplankton respiration varied from 0.01 to 0.97 μM C h^-1^, BP varied from less than 0.01–0.25 μM C h^-1^, and BGE varied from values as low as 0.01 to values as high as 0.93 (Supplementary Table S1). The overall means were 0.36 μM C h^-1^ for BR, 0.10 μM C h^-1^ for BP and 0.15 for BGE. In general, Cabiúnas and Carapebus lagoons showed higher BP rates (means = 0.16 and 0.17 μM C h^-1^, respectively) than Comprida, Amarra-Boi, and Atoleiro lagoons (means = 0.08, 0.07, and 0.03 μM C h^-1^, respectively). The Carapebus lagoon showed higher BR rates (mean = 0.45 μM C h^-1^) than the others (means = 0.37, 0.34, 0.27, and 0.30 μM C h^-1^ for Cabiúnas, Comprida, Amarra-Boi, and Atoleiro, respectively). BGE was highest in Cabiúnas lagoon (mean = 0.38), followed by Carapebus lagoon (mean = 0.29), Amarra-Boi and Comprida lagoons (means = 0.24 and 0.21, respectively), and Atoleiro lagoon (mean = 0.09; Supplementary Table S1).

**FIGURE 1:**
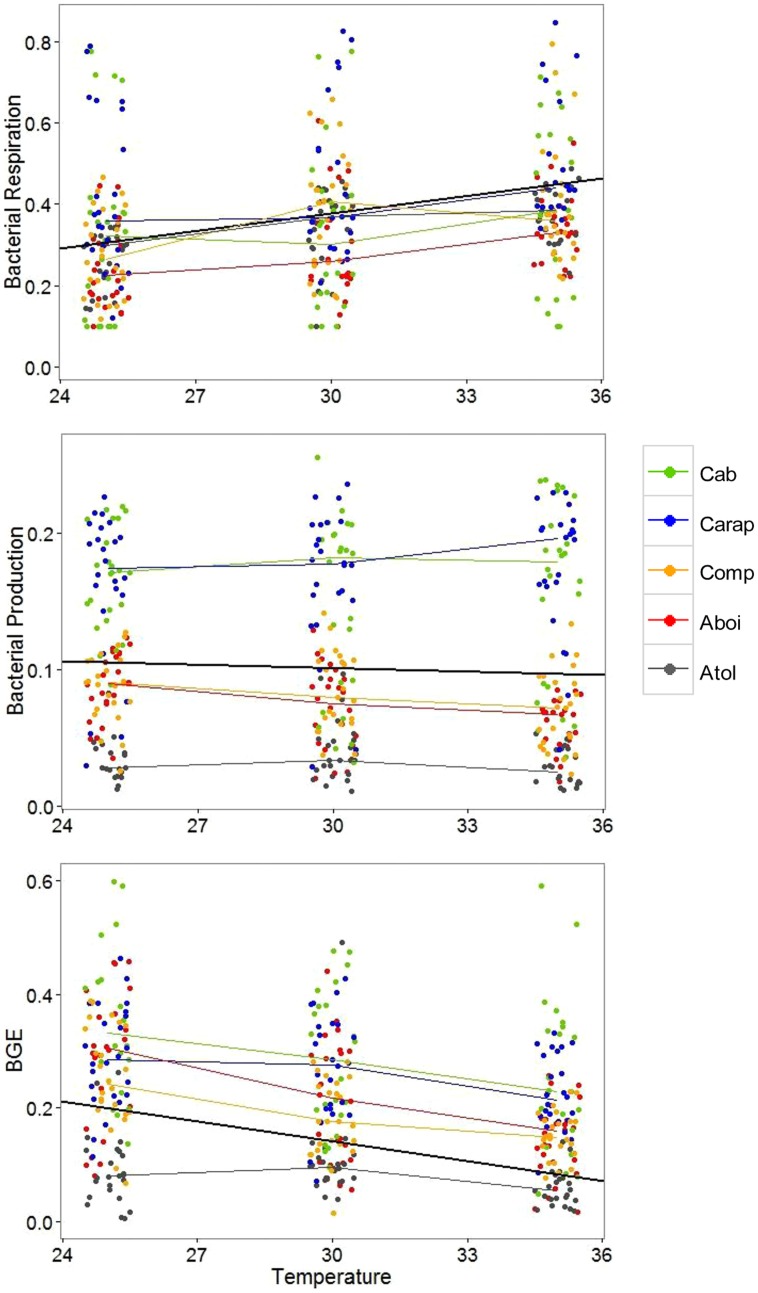
**Changes in bacterial respiration, bacterial production, and bacterial growth efficiency (BGE) in relation to the increase in water temperature in five humic tropical lagoons (Cabiunas – Cab, Carapebus – Carap, Comprida – Comp, Amarra-Boi – Aboi, and Atoleiro – Atol)**. For each temperature, all bacterial data related to different nutrient additions were pooled together because temperature effects were consistent for all lagoons and independent of nutrient additions (see **Table 2** for the analysis results). Colored thin lines link the medians of bacterial data for each lagoon at each incubation temperature. The black central line represents the best fitting line that predicts bacterial respiration (BR), production (BP), or growth efficiency (BGE) in response to water temperature. Intercepts and slopes of best fitting lines for BR, BP, or BGE are: -0.055 and 0.0144 (BR), 0.128, and -0.0009 (BP), and 0.488, slope = -0.0116 (BGE).

In the first analysis, we pooled the data from all lagoons together to observe general patterns of response to nutrient additions and increase of water temperature. Water temperature alone had the greatest effect on bacterial metabolism (**Table 2**). BR increased with increasing temperatures while BP slightly decreased with increasing temperatures. Consequently BGE decreased with increasing temperatures mainly related to the highest BR in the highest incubation temperatures. Neither nutrient additions (N or P) nor their interaction (N and P) had consistent general effects on bacterial metabolism when data from all lagoons were pooled together (i.e., there was no general pattern of nutrient limitation among lagoons, **Table 2**). The only significant interaction between water temperature and nutrient addition was observed for bacterial respiration, in which N additions interacted with the increasing water temperatures, resulting in higher BR rates. Based on these results, we established simple LMs relating bacterial metabolism to water temperature for these lagoons (**Figure 1**). An increase of about 1°C in water temperature in the lagoons lead to an increase in BR rates of approximately 12.8 nM C h^-1^, a slight decrease in BP rates of approximately 0.9 nM C h^-1^, and a decrease in BGE of approximately 0.0116.

**Table 2:** Results of linear mixed model (LMM) analysis for bacterial respiration, bacterial production and bacterial growth efficiency (BGE).

	Chi square value	*p* value
**Bacterial respiration**		
Intercept	0.38	0.706
N addition	1.52	0.128
P addition	0.21	0.832
NP additions	0.47	0.636
**Temperature (+)**	**2.76**	**0.006**
**N addition:Temperature (+)**	**2.12**	**0.034**
P addition:Temperature	0.51	0.604
NP additions:Temperature	0.17	0.864
tablegray**Bacterial production**		
**Intercept**	**3.58**	**<0.001**
N addition	1.13	0.258
P addition	0.13	0.895
NP additions	0.67	0.505
**Temperature (-)**	**2.11**	**0.035**
N addition:Temperature	1.95	0.053
P addition:Temperature	0.52	0.601
NP additions:Temperature	1.14	0.256
tablegray****Bacterial growth efficiency****		
**Intercept**	6.48	**<0.001**
N addition	0.19	0.852
P addition	0.26	0.797
NP additions	0.01	0.992
**Temperature (-)**	**4.23**	**<0.001**
N addition:Temperature	0.00	0.997
P addition:Temperature	0.45	0.651
NP additions:Temperature	0.62	0.537

*We calculated the main and interactive effects of manipulated factors (nutrient additions and temperature) on bacterial parameters for the complete dataset (all lagoons are pooled together). The block and lagoon identity were inserted as a random factors in the models. Negative or positive signals indicate a negative or positive significant effect of the variable (or interaction) on the bacterial process. Significant effects are in bold.*

**FIGURE 2:**
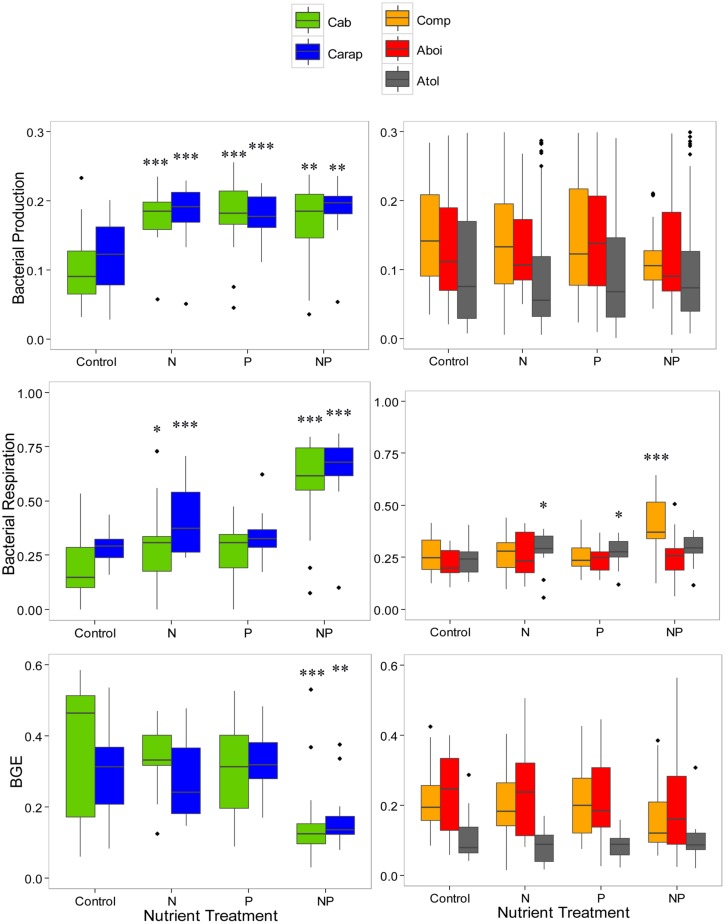
**Bacterial respiration **(upper)**, bacterial production **(middle)**, and BGE, **lower**) in relation to different nutrient additions in five humic tropical lagoons**. Lagoons were grouped in different panels based on the bacterial responses to nutrient additions. Panels on the left show data from the lagoons with lower carbon concentrations (Cabiunas – Cab, Carapebus – Carap) while panels on the right show data from the lagoons with higher carbon concentrations (Comprida, Comp, Amarra-Boi – Aboi, Atoleiro – Atol). Control – no nutrient addtion, N – nitrogen addition, P – phosphorus addition, and NP – nitrogen and phosphorus additions. Asterisks indicates a significant difference in response of bacterial metabolism to nutrient additions compared with the control for each lagoon (*p < 0.05, **p < 0.01, ***p < 0.001). Note that no comparison between lagoons is showed here.

We next analyzed the data for each lagoon separately, searching for common respoother hand, lagoons with the highest DOC concentrations (Comprida, Amarra-Boi, and Atoleiro) showed almost no significant relationship with nutrient additions: N and P additions slightly stimulated BR in Atoleiro bacterial cultures, the interaction between N and P had a strong positive effect on BR in Comprida bacterial cultures, and neither nutrient addition nor their interaction affected BP or BGE in any of these lagoons (**Figure 2**). We then pooled together the datasets for different lagoons in relation to DOC concentrations and observed that: (1) In the lagoons with the lowest DOC concentrations, BR was enhanced by the N addition and the interaction between N and P, BP was enhanced by N or P alone, and BGE decreased in response to the interaction between N and P additions, and (2) In the lagoons with the highest DOC concentrations, BR was enhanced by the N addition (**Table 3**).

**Table 3:** Results of LMM analysis for bacterial respiration, bacterial production and BGE for two partial datasets pooled in relation to dissolved organic carbon (DOC) concentration in the lagoons.

	Low DOC lagoons	High DOC lagoons
**Bacterial respiration**		
Intercept	4.817***	8.081***
N addition	3.400**	2.262*
P addition	1.731	1.228
NP interaction	4.990***	0.971
**Bacterial production**		
Intercept	13.895***	12.528***
N addition	6.269***	1.157
P addition	5.989***	0.241
NP interaction	**-**0.238	0.279
**Bacterial growth efficiency**		
Intercept	9.437***	6.207***
N addition	1.307	0.272
P addition	1.174	**-**0.764
NP interaction	**-**4.503***	**-**0.434

Cabiúnas and Carapebus lagoons were categorized as low DOC concentration lagoons, while Comprida, Amarra-Boi, and Atoleiro were categorized as high DOC concentration lagoons (see **Table 1** for DOC concentrations). We calculated the main and interactive effects of nutrient additions on bacterial parameters. Incubation temperature and lagoon identity were inserted as a random factors in the models. Negative or positive signals indicate a negative or positive significant effect of the variable (or interaction) on the bacterial process. *p < 0.05, **p < 0.01, and ***p < 0.001.

## Discussion

The main goal of this study was to evaluate how bacterioplankton metabolism and its relative importance as a carbon link or sink is singly and/or synergistically influenced by the environmental changes predicted for tropical lagoons of the Brazilian Southeast. We verified that anabolic and catabolic bacterial processes have different trends in response to temperature and nutrient manipulation, and that the strength and generality of the response varies according to the process evaluated and the factor manipulated. For instance, the increase in water temperature increased BR, decreased BP and, consequently, decreased BGE. Furthermore, the addition of nutrients did not yield a consistent change in bacterial metabolism among all lagoons, but its impact was stronger for lagoons with lower DOC concentrations. Overall, we observed few interactions between water temperature and nutrient concentration, indicating these factors almost always impacted bacterial processes independently.

An increase in the average global temperature is a major environmental change expected for this century. The Brazilian Southeast is expected an increase of temperature of 0.5–1.0°C through 2040 and of 2–3°C between 2041 and 2070 (PBMC, 2013). In temperate systems, increases in temperature are associated with an overall increase in bacterial metabolism with a positive effect on BGE (Hall et al., 2008). This effect may be direct, via increased cellular activity, or indirect, via input of nutrients from the melting of drainage basins in aquatic systems. On the other hand, temperature is consistently higher in tropical systems where tropical organisms are living close to optimum thermal conditions (Deutsch et al., 2008). Even a small increase in water temperature in tropical ecosystems is expected to drive organisms beyond optimal growing conditions (Deutsch et al., 2008), increasing energetic requirements for cell maintenance as a consequence of changes in the membrane fluidity or in the functioning of the cellular enzymatic machinery (Nedwell, 1999; Pomeroy and Wiebe, 2001). The temperature manipulations in this experiment are in the upper temperature limit found in inland waters, and are inserted in the regular temperature range found in these lagoons (Caliman et al., 2010). Therefore, the observed increase in BR and decrease in BGE related to the increase in water temperature in this experiment were expected based on known changes in bacterial physiology at high temperatures and based on the temperature range of our manipulations. In fact, higher BR rates and lower rates of carbon incorporation into bacterial biomass associated with higher temperatures were found in a survey of bacterial metabolism in tropical aquatic ecosystems (Amado et al., 2013).

As water temperature affected bacterial metabolism in all lagoons in a similar way, we established negative linear relationships between water temperature, BP and BGE, as well as a positive linear relationship between water temperature and BR. On one hand, the predictive slight negative effect of higher water temperatures on BP indicates that less carbon will be assimilated into bacterial biomass under future climate scenarios. Consequently, the microbial loop is expected to be slightly less influential as an alternative energy source for higher trophic levels in tropical aquatic ecosystems. On the other hand, the positive effect of higher temperatures on BR indicates a positive feedback between a climate change outcome (increase in average temperature) and a climate change driver (release of greenhouse gasses from natural systems). The positive and significant relationship between increasing temperature and BR has been observed previously in temperate regions, albeit, the magnitude of this effect on bacterial metabolism is relatively low compared to tropical aquatic ecosystems (Marotta et al., 2014). This marked effect of temperature in tropical inland waters is strongest in the 35°C treatment, where BR increased by 2.3–10.5-fold compared with BR in temperate aquatic ecosystems (Berggren et al., 2010; Kritzberg et al., 2010). Therefore, increases in water temperature may be more important for driving bacterial metabolism in tropical rather than temperate aquatic ecosystems, but this needs further investigation.

Bacterioplankton production is usually limited by the low P concentration in the water column of inland aquatic ecosystems (Granéli et al., 2004; Smith and Prairie, 2004; Bertoni et al., 2008), including the Carapebus and Comprida lagoons (Farjalla et al., 2002). Conversely, the nitrogen limitation of BP is far less common in inland aquatic ecosystems, yet has been observed in some Amazonian ecosystems (Rai and Hill, 1984), in the water accumulated in tank-bromeliads (Haubrich et al., 2009) and in the Cabiúnas lagoon (Farjalla et al., 2002), which is also studied here. Therefore, the observed lack of a clear change in BP in response to the addition of a specific nutrient may be associated with different limiting nutrients in different lakes. This explanation is partially corroborated by the results obtained after the dataset was divided based on DOC concentration in the lagoons: N or P stimulated BP in the lagoons with lowest DOC concentration while there was no clear pattern of change in the lagoons with highest DOC concentration. An alternative hypothesis is based on the co-limitation of BP by N and P in a given system, which may have occurred at Cabiúnas and Carapebus lagoons. However, this hypothesis is apparently contrary to the well-established Law of the Minimum (Liebig, 1843), which was first established to describe the patterns of growth limitation in individual plants but not natural communities. In natural communities, each individual or species can specifically respond to the availability of different nutrients, leading to a possible co-limitation by different nutrients of the entire community (for a review, see Harpole et al., 2011). In contrast to BP, BR was strongly influenced by N or NP additions in our study, which is an unexpected result because inorganic nutrients are more important to BP than to BR (e.g., Smith and Prairie, 2004). The nitrate added in the N and NP treatments could be anaerobically respired by denitrifying bacteria, though this scenario is unlikely because the microcosms remained under aerobic conditions throughout the experiment (data not shown) and this process would not be detected by our BR method.

The DOC concentration in the lagoons apparently influences the outcome of nutrient additions on bacterial metabolism. Ecosystems with high carbon concentrations usually show a large imbalance between nutrient availability in the system and nutrient demand by bacteria (Hessen, 1992; Jansson et al., 2006). Despite slightly higher nutrient concentration in the DOC-richest lagoons than in the DOC-poorest lagoons (**Table 1**), all lagoons showed relative greater carbon than nitrogen and phosphorus concentration (greater C:N and C:P ratios) in relation to bacterial demands (for bacterial elemental composition see Fagerbakke et al., 1996). Those ecosystems are usually characterized by strong nutrient limitation, low BP rates and low carbon conversion into CO_2_ by respiration into bacterial cells (Farjalla et al., 2009a). In this experiment, lagoons with lower carbon concentrations had stronger responses to nutrient addition than lagoons with higher carbon concentrations, which was an unexpected result because of the largest imbalance between nutrient concentration and bacterial demands found in DOC-richest lagoons. We suggest that the quality of DOC bulk for bacterial growth is inversely related to DOC concentration in these lagoons, and nutrient additions have a restricted influence in low quality DOC bulk ecosystems (Farjalla et al., 2002). Besides receiving highly humic DOC from the drainage basin, Cabiúnas and Carapebus lagoons contain extensive macrophyte stands, dominated by *Typha domingensis* and *Elecocharis interstincta*. These plants contribute high quality DOC to the water column by exuding excess organic carbon produced by photosynthesis or by leaching during the early stages of decomposition (Stepanauskas et al., 2000; Farjalla et al., 2009b). Periphytic primary production is severely limited by light availability in these coastal lagoons (Sanches et al., 2011) and light penetrates further down the water column of Cabiunas and Carapebus lagoons than in the highly humic lagoons. On the other hand, macrophyte stands are restricted or absent and autochthonous primary production is very low in Comprida, Amarra-Boi, and Atoleiro lagoons. The input of DOC is exclusively external via the drainage basin, and its composition is almost exclusively of highly refractory humic compounds. Therefore, the increase in bacterial metabolism after nutrient addition in the lowest DOC concentration lagoons was most likely related to the ease of consumption and assimilation of more labile autochthonous DOC compounds, while in the highest DOC concentration lagoons the refractory characteristics of the DOC bulk restricted the responses of bacterial metabolism after nutrient additions, at least over the duration of the experiment (48 h).

Some peculiarities of the experimental design may have influenced our results. For example, some delayed physiological and re-growth responses of bacterial communities related to the treatments could have occurred and been observed under longer incubation periods than those used in our experiment. Thus, our results represent only a snapshot of rapid responses of bacterial metabolism to changes in environmental conditions. Follow-up studies focused on longer incubation periods should be performed to determine whether these responses would be maintained for long time periods. Changes in the bacterial community composition during the experiment related to the different treatments may have occurred; however, as such changes were previously observed in longer experiments, we did not expect any significant effect here. The spatiotemporal representation of the study may have been compromised, as we sampled only once from each lagoon. Nonetheless, we have observed in previous studies that the greatest changes in bacterial processes occur between lagoons, not within the same lagoon over time or between sampling stations in the same lagoon (MacCord et al., 2013). The use of artificial microcosms was necessary for a thorough evaluation without the confounding effects of varying the concentrations of nutrients and water temperature on bacterial metabolism; regardless, extrapolation from studies in artificial microcosms to natural systems should be performed with caution. It is noteworthy that the nutrient concentrations used in this study were the same as those observed in the actual lagoons or in similar systems with domestic sewage input (Caliman et al., 2010). Finally, although an increase of 10°C is not predicted in any future climate scenario, this change in water temperature is usually found throughout a single day in summer months in shallow lagoons (Farjalla et al., 2005). The impact of these episodic events on long term microbial metabolism and carbon cycling in these ecosystems remain to be evaluated.

We conclude that increase in water temperature impacted microbial metabolism in the tropical coastal lagoons by increasing BR, slightly decreasing BP, and decreasing BGE. Nutrient inputs also changed the bacterial metabolism, but the magnitude of alterations varied among lagoons and seemed to be related to the availability and quality of carbon substrates in each system. Overall, both nutrient additions and increases in water temperature decreased BGE. This result points to important changes in the future carbon cycle of aquatic ecosystems related to the role of bacterioplankton. In the future, it is expected that the predicted environmental changes will favor processes related to bacterial catabolism, whereby relatively more carbon is converted into CO_2_ than into bacterial biomass available to higher trophic levels through the microbial loop.

## Conflict of Interest Statement

The authors declare that the research was conducted in the absence of any commercial or financial relationships that could be construed as a potential conflict of interest.
