# Supplementary material for: Potential changes in bacterial metabolism associated with increased water temperature and nutrient inputs in tropical humic lagoons
Source: Front Microbiol. 2015 Apr 15;6:310. doi: 10.3389/fmicb.2015.00310 (PMC4397971; doi:10.3389/fmicb.2015.00310)
Supplement: Supplementary file 1 [file Table_1.DOCX]

**Supplementary Material**

Table S1. Mean, standard deviation and range (minimum-maximim) of (A) bacterial respiration (µM C h^-1^), (B) production (µM C h^-1^), and (C) growth efficiency in the four nutrient treatments crossed with the three water temperature incubations, in the five studied tropical coastal lagoons.

(A)

|  | Cabiúnas Lagoon | | | Carapebus Lagoon | | | Comprida Lagoon | | | | | | | |
| --- | --- | --- | --- | --- | --- | --- | --- | --- | --- | --- | --- | --- | --- | --- |
|  | 25 °C | 30 °C | 35 °C | 25 °C | 30 °C | 35 °C | 25 °C | | | 30 °C | | | 35 °C | |
| Control | 0.17±0.15 (0.01-0.36) | 0.23±0.13 (0.09-0.43) | 0.30±0.24 (0.09-0.64) | 0.30±0.07 (0.19-0.38) | 0.31±0.05 (0.26-0.37) | 0.42±0.06 (0.34-0.52) | 0.28±0.11 (0.15-0.40) | | | 0.35±0.12 (0.18-0.50) | | | 0.30±0.08 (0.22-0.42) | |
| N addition | 0.20±0.16 (0.01-0.38) | 0.25±0.18 (0.03-0.40) | 0.53±0.22 (0.26-0.88) | 0.40±0.15 (0.29-0.63) | 0.45±0.16 (0.29-0.68) | 0.63±0.19 (0.39-0.85) | 0.23±0.09 (0.12-0.34) | | | 0.36±0.11 (0.20-0.52) | | | 0.37±0.09 (0.27-0.53) | |
| P addition | 0.25±0.19 (0.01-0.42) | 0.31±0.13 (0.15-0.49) | 0.34±0.22 (0.01-0.57) | 0.34±0.08 (0.23-0.42) | 0.38±0.11 (0.21-0.53) | 0.48±0.14 (0.36-0.74) | 0.27±0.08 (0.17-0.36) | | | 0.28±0.11 (0.17-0.45) | | | 0.36±0.10 (0.28-0.52) | |
| NP additions | 0.56±0.27 (0.09-0.78) | 0.69±0.25 (0.23-0.92) | 0.85±0.13 (0.64-0.95) | 0.61±0.25 (0.12-0.79) | 0.82±0.07 (0.74-0.91) | 0.92±0.04 (0.87-0.97) | 0.35±0.13 (0.15-0.47) | | | 0.56±0.09 (0.45-0.66) | | | 0.56±0.19 (0.37-0.79) | |
|  | Amarra-Boi Lagoon | | | Atoleiro Lagoon | | | |  | | |  | | |  |
|  | 25 °C | 30 °C | 35 °C | 25 °C | 30 °C | 35 °C | | |  | | |  | | |
| Control | 0.24±0.09 (0.13-0.37) | 0.24±0.09 (0.13-0.35) | 0.30±0.07 (0.22-0.40) | 0.23±0.06 (0.16-0.31) | 0.28±0.11 (0.18-0.44) | 0.35±0.09  (0.23-0.49) | | |  | | |  | | |
| N addition | 0.26±0.14 (0.13-0.45) | 0.31±0.13 (0.20-0.48) | 0.38±0.12 (0.25-0.55) | 0.30±0.06 (0.17-0.34) | 0.35±0.14 (0.07-0.44) | 0.40±0.05  (0.33-0.46) | | |  | | |  | | |
| P addition | 0.24±0.06 (0.17-0.30) | 0.28±0.06 (0.22-0.36) | 0.35±0.08 (0.22-0.47) | 0.27±0.08 (0.14-0.35) | 0.36±0.06 (0.29-0.44) | 0.37±0.06  (0.30-0.45) | | |  | | |  | | |
| NP additions | 0.21±0.09 (0.09-0.31) | 0.34±0.19 (0.08-0.61) | 0.34±0.05 (0.25-0.41) | 0.28±0.08 (0.14-0.35) | 0.38±0.07 (0.27-0.45) | 0.40±0.05  (0.34-0.45) | | |  | | |  | | |

Cont.

(B)

|  | Cabiúnas Lagoon | | | Carapebus Lagoon | | | Comprida Lagoon | | | | | |
| --- | --- | --- | --- | --- | --- | --- | --- | --- | --- | --- | --- | --- |
|  | 25 °C | 30 °C | 35 °C | 25 °C | 30 °C | 35 °C | 25 °C | | 30 °C | | 35 °C | |
| Control | 0.12±0.02  (0.09-0.14) | 0.06±0.02  (0.03-0.09) | 0.12±0.07  (0.06-0.23) | 0.13±0.06  (0.03-0.19) | 0.10±0.06  (0.03-0.16) | 0.12±0.06  (0.04-0.20) | 0.09±0.02  (0.06-0.12) | | 0.09±0.04  (0.04-0.14) | | 0.06±0.02  (0.03-0.09) | |
| N addition | 0.17±0.03  (0.15-0.22) | 0.17±0.05  (0.05-0.20) | 0.19±0.04  (0.15-0.23) | 0.17±0.06  (0.05-0.21) | 0.18±0.03  (0.13-0.22) | 0.20±0.04  (0.14-0.24) | 0.08±0.03  (0.05-0.12) | | 0.07±0.04  (0.01-0.11) | | 0.07±0.02  (0.04-0.09) | |
| P addition | 0.18±0.03  (0.15-0.22) | 0.17±0.07  (0.04-0.25) | 0.17±0.05  (0.08-0.24) | 0.17±0.04  (0.11-0.21) | 0.19±0.03  (0.15-0.22) | 0.17±0.04  (0.11-0.20) | 0.08±0.03  (0.03-0.11) | | 0.07±0.02  (0.04-0.11) | | 0.06±0.03  (0.02-0.09) | |
| NP additions | 0.18±0.04  (0.11-0.21) | 0.18±0.04  (0.13-0.20) | 0.15±0.09  (0.04-0.24) | 0.17±0.06  (0.05-0.22) | 0.20±0.03  (0.15-0.23) | 0.19±0.04  (0.16-0.22) | 0.09±0.03  (0.05-0.12) | | 0.09±0.03  (0.06-0.13) | | 0.10±0.03  (0.04-0.13) | |
|  | Amarra-Boi Lagoon | | | Atoleiro Lagoon | | | |  | |  | |  |
|  | 25 °C | 30 °C | 35 °C | 25 °C | 30 °C | 35 °C |  | |  | |  | |
| Control | 0.09±0.02  (0.06-0.11) | 0.06±0.04  (0.02-0.10) | 0.05±0.01  (0.03-0.07) | 0.03±0.03  (0.01-0.08) | 0.02±0.00  (0.02-0.03) | 0.02±0.01  (0.01-0.04) |  | |  | |  | |
| N addition | 0.08±0.03  (0.05-0.12) | 0.09±0.02  (0.05-0.11) | 0.07±0.01  (0.05-0.09) | 0.02±0.02  (0.00-0.05) | 0.06±0.03  (0.03-0.09) | 0.02±0.01  (0.01-0.04) |  | |  | |  | |
| P addition | 0.09±0.03  (0.05-0.12) | 0.07±0.04  (0.03-0.13) | 0.05±0.03  (0.01-0.08) | 0.02±0.01  (0.00-0.03) | 0.04±0.01  (0.02-0.05) | 0.03±0.02  (0.01-0.05) |  | |  | |  | |
| NP additions | 0.07±0.04  (0.03-0.12) | 0.06±0.02  (0.03-0.08) | 0.05±0.03  (0.01-0.08) | 0.04±0.01  (0.02-0.05) | 0.04±0.02  (0.01-0.07) | 0.03±0.01  (0.01-0.04) |  | |  | |  | |

(C)

|  | Cabiúnas Lagoon | | | Carapebus Lagoon | | | Comprida Lagoon | | | | | |
| --- | --- | --- | --- | --- | --- | --- | --- | --- | --- | --- | --- | --- |
|  | 25 °C | 30 °C | 35 °C | 25 °C | 30 °C | 35 °C | 25 °C | | 30 °C | | 35 °C | |
| Control | 0.53±0.31 (0.20-0.92) | 0.25±0.12 (0.13-0.42) | 0.36±0.22 (0.08-0.64) | 0.30±0.13 (0.11-0.46) | 0.24±0.13 (0.07-0.38) | 0.22±0.09 (0.09-0.33) | 0.27±0.08 (0.19-0.39) | | 0.21±0.05 (0.14-0.26) | | 0.16±0.05 (0.09-0.23) | |
| N addition | 0.57±0.29 (0.31-0.93) | 0.48±0.21 (0.32-0.85) | 0.28±0.07 (0.19-0.37) | 0.30±0.10 (0.14-0.43) | 0.30±0.07 (0.20-0.38) | 0.25±0.03 (0.21-0.30) | 0.27±0.08 (0.18-0.36) | | 0.16±0.09 (0.02-0.28) | | 0.16±0.03 (0.13-0.20) | |
| P addition | 0.53±0.32 (0.28-0.93) | 0.37±0.19 (0.10-0.63) | 0.42±0.29 (0.12-0.93) | 0.33±0.05 (0.28-0.38) | 0.35±0.06 (0.28-0.43) | 0.27±0.05 (0.19-0.31) | 0.25±0.11 (0.07-0.39) | | 0.21±0.07 (0.12-0.28) | | 0.13±0.04 (0.08-0.19) | |
| NP additions | 0.30±0.21 (0.14-0.70) | 0.23±0.13 (0.12-0.48) | 0.14±0.06 (0.05-0.20) | 0.24±0.04 (0.21-0.31) | 0.19±0.01 (0.17-0.21) | 0.17±0.02 (0.14-0.20) | 0.23±0.10 (0.09-0.35) | | 0.14±0.05 (0.09-0.23) | | 0.15±0.05 (0.09-0.23) | |
|  | Amarra-Boi Lagoon | | | Atoleiro Lagoon | | | |  | |  | |  |
|  | 25 °C | 30 °C | 35 °C | 25 °C | 30 °C | 35 °C |  | |  | |  | |
| Control | 0.29±0.07 (0.16-0.36) | 0.22±0.13 (0.06-0.35) | 0.15±0.06 (0.08-0.24) | 0.12±0.08 (0.04-0.26) | 0.08±0.02 (0.06-0.10) | 0.05±0.02  (0.03-0.08) |  | |  | |  | |
| N addition | 0.28±0.15 (0.10-0.46) | 0.25±0.10 (0.10-0.33) | 0.17±0.06 (0.10-0.24) | 0.06±0.05 (0.02-0.13) | 0.19±0.20 (0.07-0.59) | 0.05±0.03  (0.02-0.11) |  | |  | |  | |
| P addition | 0.29±0.10 (0.14-0.41) | 0.21±0.09 (0.11-0.34) | 0.13±0.08 (0.02-0.26) | 0.06±0.03 (0.01-0.08) | 0.10±0.03 (0.04-0.14) | 0.07±0.05  (0.02-0.14) |  | |  | |  | |
| NP additions | 0.28±0.17 (0.08-0.47) | 0.19±0.16 (0.08-0.51) | 0.13±0.08 (0.02-0.18) | 0.13±0.06 (0.08-0.24) | 0.09±0.04 (0.04-0.15) | 0.06±0.03  (0.02-0.09) |  | |  | |  | |
